# Supplementary material for: Content-rich biological network constructed by mining PubMed abstracts
Source: BMC Bioinformatics. 2004 Oct 8;5:147. doi: 10.1186/1471-2105-5-147 (PMC528731; doi:10.1186/1471-2105-5-147)
Supplement: Additional File 2 — The original results of the above study (non-essential files are deleted to keep the file size under the limit set by BMC bioinformatics). [file 1471-2105-5-147-S2.bz2 › chilibotAdditionalFile2/dip05/513ID10344732E10815/html/CCND1_AR.html]

 


 **CCND1** and **AR** 
  
Found 122 abstracts in PubMed, retrieved 05.  
 

 What does Google say? 
 PDF only 
| .edu only 

---

**Interactive relationship** (e.g. stimulation, inhibition, etc)

**Inhibitory relationship**- Pretreatment with PD98059 suppressed  **AR**  induced cyclin D1  [ **CCND1** ]  mRNA and protein.  Ref: 12869389 Circ ResCirc Res,
**Stimulatory relationship**- In contrast,  **AR** , a specific ErbB1 ligand, induced sustained ERK1 2 and Elk1 phosphorylation, increased cyclin D1  [ **CCND1** ]  mRNA and protein, and increased DNA synthesis activity.  Ref: 12869389 Circ ResCirc Res,
- Treatment of LY  **ar**  cells with the MEK inhibitors PD 98059, U0126, and PD 184352 led to a loss of phosphorylated ERK1 and ERK2, a reversal of nuclear p50 homodimer DNA binding, and a decrease in Bcl  [ **CCND1** ]  2 protein expression.  Ref: 12801933 J Biol Chem< MedlineTA>J Biol Chem,
**Neutral relationship**- ... a sustained ERK Elk1 activation could be the major signaling cascade for  **AR**  induced upregulation of cyclin D1  [ **CCND1** ]  protein in HTASMCs.  Ref: 12869389 Circ ResCirc Res,

**Non-interactive relationship** (e.g. studied together, co-existance, homology, etc.)

- We examined the impact of beta  **AR**  blockade with metoprolol on myocardial remodeling, apoptosis, pro apoptotic Fas, Fas ligand, Bax, and Bcl  [ **CCND1** ]  X S and anti apoptotic Bcl  [ **CCND1** ]  X L and Bcl  [ **CCND1** ]  2 gene expression, and Bcl  [ **CCND1** ]  X L and Bcl  [ **CCND1** ]  X S protein in post infarction HF in rats.  Ref: 12738230 J Mol Cell Cardiol, 2003
- In a previously published report, we described the NF kappaB status for two murine B cell lymphoma cell lines, LY as apoptosis sensitive and LY  **ar**  apoptosis refractory, and provided evidence that NF kappaB1 p50 homodimers contribute to the expression of Bcl  [ **CCND1** ]  2 in the LY  **ar**  line.  Ref: 12801933 J Biol Chem< MedlineTA>J Biol Chem, 2003
- We conclude that adrenergic activation, such as occurs in HF, increases pro apoptotic Bcl  [ **CCND1** ]  X S expression via the beta 1  **AR** . beta  **AR**  blockade in HF reduces myocardial apoptosis.  Ref: 12738230 J Mol Cell Cardiol, 2003
